# Renal maturation and catch-up clearance of ganciclovir in a preterm neonate: Bayesian pharmacokinetic analysis using a population model

**DOI:** 10.1186/s40780-025-00496-5

**Published:** 2025-10-21

**Authors:** Katsuya Kata, Satomi Inomata, Mitsuru Nishikawa, Haruka Ide, Kentaro Nakamura, Taketoshi Yoshida, Masato Taguchi

**Affiliations:** 1 Department of Pharmacy Practice and Sciences, School of Pharmacy and Pharmaceutical Sciences, University of Toyama, 2630 Sugitani, Toyama, 930-0194 Japan; 2 Division of Neonatology, Maternal and Perinatal Center, Toyama University Hospital, Toyama, Japan

**Keywords:** Ganciclovir, Valganciclovir, Developmental, Maturation, Preterm, Low birth weight, Infants, PPK

## Abstract

**Background:**

Congenital cytomegalovirus (CMV) infection is a major cause of neonatal morbidity. However, optimizing ganciclovir (GCV) dosing in preterm infants is complicated by immature renal function and developmental pharmacokinetics. Population pharmacokinetic (PPK) parameters for GCV and valganciclovir (VGCV) have been reported, but data remain limited for extremely low birth weight infants. We aimed to characterize longitudinal changes in GCV clearance in a preterm infant with congenital CMV infection using Bayesian modeling.

**Methods:**

GCV/VGCV were administered over a 15-week period, with concurrent therapeutic drug monitoring. Individual parameters were estimated using a previously published PPK model and a postnatal age-based maturation function in NONMEM. Scr clearance was measured at two time points using the 24-h urine collection method.

**Results:**

GCV clearance increased from 0.048 to 0.273 L/hr/kg from postnatal day 30 to day 93, whereas VGCV bioavailability remained stable (~ 52–55%). Scr clearance values matched estimated GCV clearance, supporting the validity of the model. The maturation function indicated that tubular secretion likely contributes to accelerated drug elimination. Late-phase GCV clearance exceeded typical glomerular filtration rate, indicating possible catch-up in renal function.

**Conclusion:**

Renal maturation should be considered in addition to body weight when adjusting GCV dosing in preterm infants. This case highlights the importance of aligning individualized dosing strategies with the developmental physiology of preterm neonates.

## Background

Congenital cytomegalovirus (CMV) infection is the most common congenital infection and is classified as part of the TORCH syndrome group. Fetal infection occurs through transplacental transmission from the infected mother [1]. A large-scale screening survey using urine samples from more than 21,000 newborns conducted in Japan found congenital CMV infection in approximately 1 in 300 births (0.31%) [2]. Moreover, 10–15% of infants with congenital CMV infection are symptomatic, and the mortality rate during the neonatal period is 30%. Sequelae, such as developmental delay and hearing loss, occur in 40–80% of symptomatic and 10–15% of asymptomatic infants [3–5]. Therefore, therapeutic intervention is necessary.

Ganciclovir (GCV) is used to treat congenital CMV. It is selectively activated in virus-infected cells and inhibits viral DNA polymerase [6]. GCV is administered intravenously due to its low oral bioavailability (approximately 6%) [7]. Valganciclovir (VGCV), an L-valyl ester prodrug of GCV, was developed to improve bioavailability. After oral absorption, VGCV is rapidly converted to GCV, which is then mainly excreted unchanged in the urine [8]. Acosta et al. [9] reported the population pharmacokinetics (PPK) of oral VGCV in neonates with congenital CMV infection. The typical bioavailability (F) was estimated to be 53.6%. GCV clearance (CL) and volume of distribution (V) were described by the equations CL = $0.146\cdot W{T}^{1.18}$ (L/h) and V= $1.15\cdot WT$ (L), where WT is body weight in grams.

According to the treatment protocol for congenital CMV infection [10], the dose of GCV is 6 mg/kg twice daily for infants meeting the following criteria: gestational age > 32 weeks, body weight > 1,200 g at birth, and postnatal age (PNA) < 30 days. However, a dosing regimen for preterm infants who do not meet these criteria is challenging due to difficulties in accurately assessing renal function using serum creatinine, cystatin C levels, or the Schwartz formula in this population; therefore, the optimal mean dosage remains to be established. Renal function in preterm infants develops rapidly over a short period. In the most immature group of infants, a previous study reported that creatinine clearance (CLcr) increased from 0.65 to 1.73 mL/min within two weeks [11].

We experienced a case of an extremely low birth weight premature infant with congenital CMV infection. GCV/VGCV doses were adjusted over a period of approximately 15 weeks while measuring blood levels of GCV. This study aimed to elucidate the longitudinal changes in GCV pharmacokinetics over the course of treatment. We performed a retrospective study using Bayesian estimation with PPK parameters from a previous study [9].

## Case presentation

A female infant weighing 556 g was delivered via cesarean section at a gestational age of 27 weeks and 0 days due to non-reassuring fetal status. The mother tested positive for CMV IgM during pregnancy, and congenital CMV infection in the neonate was confirmed by detection of CMV DNA in urine during the early neonatal period.

**Fig. 1:**
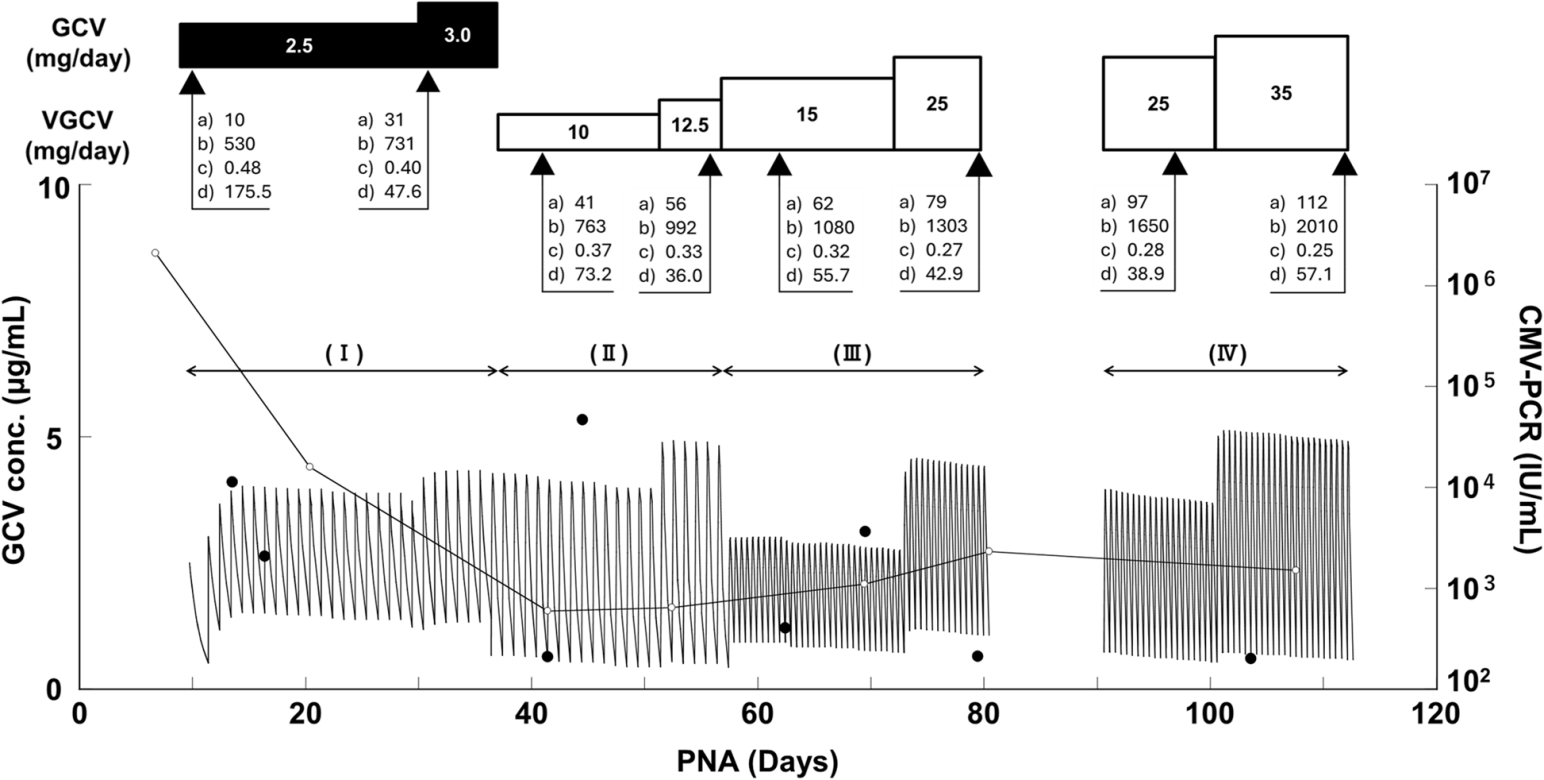
Plasma concentration of GCV and CMV-PCR during the treatment period. Closed circles (●) and open circles (○) represent plasma concentrations of GCV and CMV-PCR, respectively. Black and white bars represent the daily doses of GCV and VGCV during the treatment period. Clinical data include a) shows postnatal age (days), b) shows body weight (g), c) shows serum creatinine level (mg/dL), and d) shows white blood cell (WBC) count (× 10^2^/μL). The treatment period is divided into four phases (I–IV). Drug administration was temporarily discontinued between PNA 79 and PNA 91

Figure 1 shows the course of GCV therapy, including dosing amounts of GCV and VGCV, as well as the following key clinical laboratory parameters: PNA (days), body weight (g), serum creatinine levels (mg/dL), and white blood cell count (× 10^2^/μL). GCV (DENOSINE® for I.V. Infusion) was initiated at 2.5 mg/day once daily (s.i.d.) on PNA 9. When the infant’s weight reached 731 g, the dosage was increased to 3.0 mg/day (s.i.d.). Starting from PNA 36, VGCV (VALIXA® Dry syrup) was administered at 10 mg/day (s.i.d.), subsequently increased to 12.5 mg/day (s.i.d.), 15 mg/day twice daily (b.i.d.) from PNA 57, 25 mg/day (b.i.d.), and 35 mg/day (b.i.d.). VGCV was temporarily discontinued at PNA 79 due to neutropenia, resumed at PNA 91, and then discontinued again at PNA 112. The magnitude of CMV-PCR decreased from 2.1 × 10^6^ IU/mL prior to initiation of GCV therapy to 5.9 × 10^2^ IU/ml by PNA 41, then reached a plateau (Fig. 1).

## Methods

### Reagents

GCV powder fabricated by Tokyo Kasei (p/n G0315) was used. Acetonitrile, methanol, distilled water of HPLC-grade, and other chemicals of analytical-grade were supplied by FUJIFILM Wako Pure Chemical Corporation (Osaka, Japan).

### Sample collection

Blood samples were collected immediately before GCV administration (trough) and 2 h post-administration (peak). Urine samples were collected using the 24-h urine method on PNA 6 and PNA 52. All samples were stored at −30 °C until analysis.

### GCV assay

Plasma concentrations of GCV were determined using the previously established HPLC method with minor modifications [12]. Briefly, 50 μl of plasma samples was deproteinized with 50 μl of 1% perchloric acid, 50 μl of the mobile phase was added, and it was centrifuged at 13,500 rpm for 10 min at 4 °C. Then, 100 μl of the supernatant mixed with 50 μl of 0.04 M NaOH was injected into the HPLC system. The HPLC system consisted of a Shimadzu LC‐10ATVP (Shimadzu, Kyoto, Japan), a Cosmosil® 5C18 reversed‐phase column (Nakalai tesque, Kyoto, Japan) (4.6 × 150 mm), and a model RF‐20A fluorescence detector (Shimadzu, Kyoto, Japan). Separation of GCV was performed isocratically using sodium phosphate buffer (pH 2.5; 25 mM) containing a 1% mixed solvent of methanol–acetonitrile (4:3, v/v) at a flow rate of 0.7 ml/min, and a column temperature of 40℃. The detection wavelengths were 265 nm (λex) and 380 nm (λem) for GCV. A linear calibration range of 0.1 to 5.0 μg/mL was established and found adequate for quantifying GCV levels. The inter-day precision (coefficients of variation) was 2.2% and 0.06% at a GCV plasma concentration of 0.1 µg/ml and 5 µg/ml, respectively. The detection limit for GCV was a plasma concentration of 0.05 µg/ml.

### Pharmacokinetic analysis

Bayesian estimation was performed using the PPK model and previously reported parameters [9]. In brief, intravenous doses of GCV were administered directly into the central compartment, while oral VGCV doses were administered into an absorption depot compartment. VGCV absorption was modeled as a first-order process, with instantaneous conversion to GCV occurring in the central compartment. A one-compartment pharmacokinetic model with first-order elimination for GCV disposition was specified with ADVAN2 and TRANS2. Inter-individual variability for clearance (CL) and bioavailability (F) was captured by exponential models. Residual unexplained variability was also assumed to be log normal. The estimation method implemented for all runs was the first-order conditional estimation with interaction (FOCE-I). CL, V, absorption rate constant (Ka), and F are described by the following equation [9].

1 $$CL=0.146\cdot WT^{1.68}\left(L/h\right)$$

2 $$V=1.15\left(L/kg\right)$$

3 $$Ka=0.591\left(h^{-1}\right)$$

4 $$F=0.536$$

WT is individual body weight in kg and 1.68 is the allometric exponent derived from previous pharmacokinetic studies [9].

NONMEM version 7.5.1 were used for the Bayesian analysis [13].

### Maturation analysis

The maturation of GCV clearance was described by using a sigmoid hyperbolic or Hill model, represented by the following equation [14].

5 $$CL=\theta_1+\theta_2\cdot\frac{PNA^{\theta_3}}{\theta_4^{\theta_3}+PNA^{\theta_3}}\left(mL/min/1.73m^2\right)$$

Where PNA represents postnatal age, and $${\theta }_{1}$$, $${\theta }_{2}$$, $${\theta }_{3}$$, and $${\theta }_{4}$$ are the model parameters to be estimated. $${\theta }_{1}$$ is the intercept of this curve, which corresponds to the GCV clearance at PNA 0, as maturation of CL begins before birth. $${\theta }_{4}$$ describes the maturation half-time, whereas $${\theta }_{3}$$ (Hill coefficient) describes the slope of maturation. The variability was modeled using a proportional error model incorporating a random variable η, which follows a normal distribution with mean 0 and variance $${\omega }^{2}$$. The 95% confidence intervals (CIs) of the parameters were calculated from the standard error (SE) values estimated for all parameters: $$95\% CI = (estimated parameter value) \pm 1.96 \times SE)$$. NONMEM version 7.5.1 was used for data analysis [13].

### CMV DNA assay

Blood CMV DNA levels were monitored using a real time PCR assay, which was outsourced to and conducted by SRL, Inc. (Tokyo, Japan).

### Estimation of creatinine clearance from serum creatinine (Scr)

The Scr value was calculated using the 24-h urine method and normalized to a body surface area (BSA) of 1.73 m^2^. The calculation was based on the following formula:

6 $$Scr\;clearance=\frac{\left(Cru\cdot Vu/Scr\cdot t\right)}{\left(1.73/BSA\right)}\left(mL/min/1.73m^2\right)$$

where Cr_u_ is urine creatinine concentration (mg/dL), V_u_ is total urine volume (mL), S_cr_ is serum creatinine concentration (mg/dL), t is sampling time (min), and BSA is body surface area (m^2^).

## Result

Figure 1 shows observed and estimated plasma GCV concentrations during the treatment period. The target of the peak plasma GCV concentration was set at 5 μg/mL for this case, and observed peak concentrations of GCV were 4.09, 5.34, and 3.12 μg/ml at PNA 13, PNA 44, and PNA 69, respectively. The trough concentrations of GCV were 2.63, 0.64, 1.21, 0.65, and 0.60 g/ml at PNA 16, PNA 41, PNA 62, PNA 79, and PNA 104, respectively. The patient’s body weight increased from 530 to 2,010 g during the treatment (Fig. 1).

Bayesian analysis did not yield individual parameter estimates when the observed GCV data over the entire treatment period were analyzed as a single individual. Considering that the patients were growing and the dosage per body weight increased, the treatment period was divided into four terms. Phase I was from PNA 9 to PNA 35, when the patient was administrated GCV intravenously. The period from PNA 36 to PNA 79 was divided into Phase II and Phase III due to weight gain during this interval. Phase IV was from PNA 91 to PNA 112, which is the duration after the resumption of VGCV treatment (Fig. 1). Table 1 summarizes the pharmacokinetic parameters estimated for each phase. GCV clearance normalized to body weight increased from 0.048 L/hr/kg (PNA 30) to 0.273 L/hr/kg (PNA 93), whereas the volume of distribution (V) remained constant at 1.15 L/kg throughout the treatment. In addition, F values of VGCV were estimated as 55.0, 52.4, and 52.3% during Phases II, III, and IV, respectively (Table 1).

**Table 1:** Pharmacokinetic parameters of GCV and VGCV

PNA (days)	30	51	72	93
CL (L/hr/kg)	0.048	0.134	0.208	0.273
CL/F (L/hr/kg)	ー	0.244	0.397	0.522
V (L/kg)	1.15	1.15	1.15	1.15
F (%)	ー	55.0	52.4	52.3

*PNA* postnatal ages, *CL* clearance, *CL/F* oral clearance, *V* distribution volume, *F* bioavailability

**Fig. 2:**
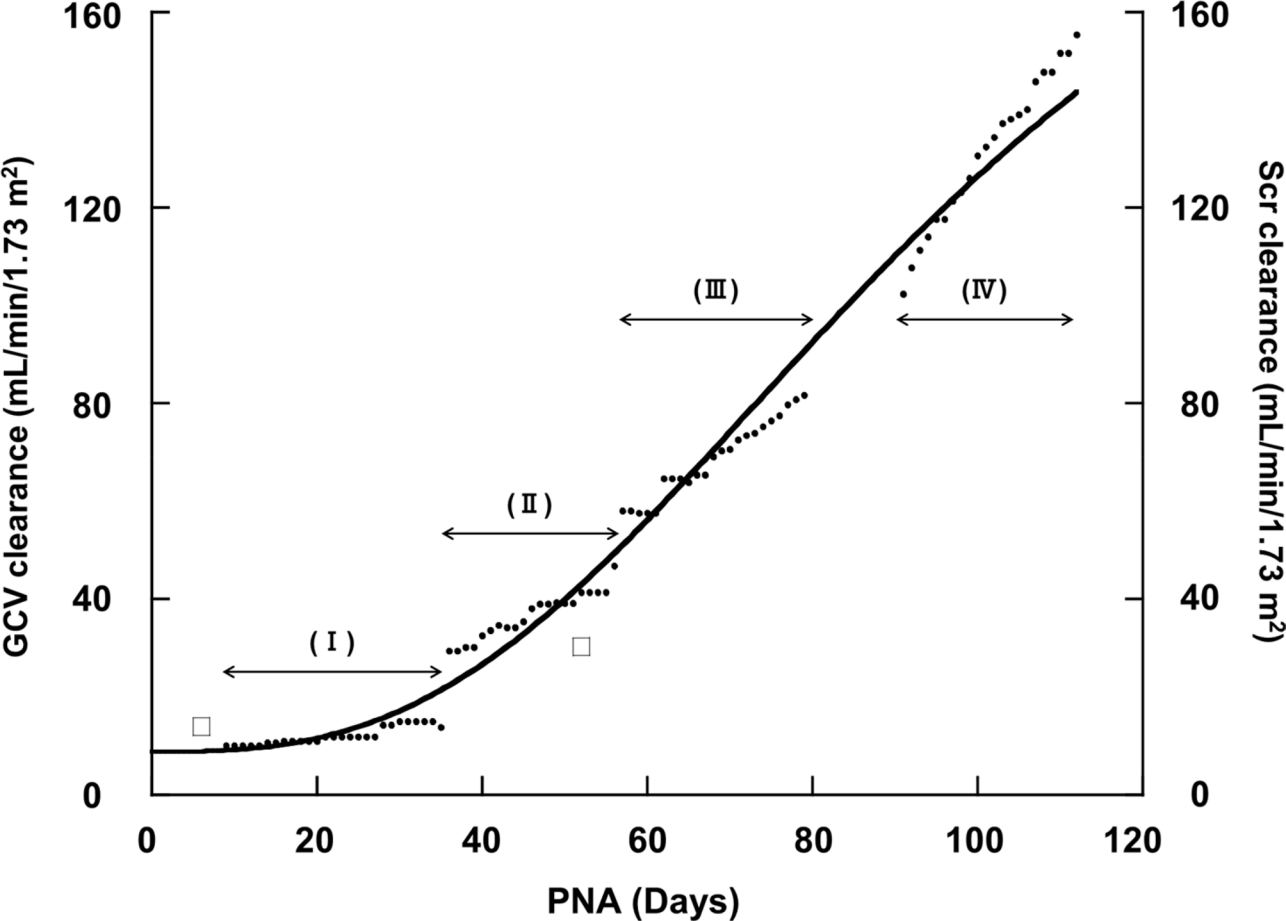
Developmental changes in GCV clearance and Scr clearance values. Black dots are estimated values of GCV clearance. The solid line represents the maturation model of GCV clearance described using the Hill function. Open squares are Scr clearance values at days 6 and 52 determined by the 24-h urine method. The treatment period is divided into four phases (I–IV). Drug administration was temporarily discontinued between PNA 79 and PNA 91

Table 2 shows the estimated parameters of the maturation model of GCV clearance. CL values were normalized to body surface area (mL/min/1.73m^2^): ${\theta }_{1}$ was 8.80; ${\theta }_{2}$ was 222; ${\theta }_{3}$, which represents the Hill coefficient, was 2.79; and ${\theta }_{4}$, which is the age at 50% maturity of GCV CL, was 95.8 (days) (Table 2). Fig. 2 shows the modeled increase in GCV clearance over time and a comparison with the estimated creatinine clearance (Scr clearance). Two data points, indicated by open squares, were determined by the 24-h urine method, thus providing accurate reference values (Fig. 2). These empirical measurements are regarded as true values and demonstrated close agreement with the estimated GCV clearance, thereby supporting the validity of the pharmacokinetic model. A noteworthy finding was that GCV clearance in the late treatment period exceeded 100 mL/min/1.73m^2^, which approximates the normal glomerular filtration rate.

**Table 2:** Parameter estimates of the maturation model

Parameter	Parameter estimates	95% CI
θ_1_^a)^	8.80	8.26–9.34
θ_2_^a)^	222	169–274
θ_3_	2.79	2.43–3.15
θ_4_^b)^	95.8	79.5–112
ω^2^	0.0126	0.00817–0.0170

The θ_2_, θ_3_, and θ_4_ represent the scaling factor, Hill coefficient, and the maturation half-time, respectively. The ω^2^ is an interindividual variance

^a)^mL/min/1.73m^2^, ^b)^days, *CI* confidence interval

## Discussion

We retrospectively analyzed the pharmacokinetics of GCV and VGCV in a preterm infant born at 27 weeks of gestation with a birth weight of 556 g, with a focus on estimating CL values using the Bayesian approach. The GCV clearance increased from 0.048 L/hr/kg at PNA 30 to 0.273 L/hr/kg at PNA 93 (Table 1, Fig. 1). The two measured values of Scr clearance were in good agreement with GCV clearance at each point. The developmental changes in GCL clearance were well approximated by a maturation model (Table 2). Notably, the GCV clearance surpassed Scr clearance during the late treatment phase (Fig. 2).

The mechanism underlying GCV clearance exceeding CLcr remains unclear, although the observed difference suggests the involvement of active tubular secretion. Supporting this hypothesis, Takeda et al. [15] reported that renal transport of GCV involves human organic anion transporter 1 (hOAT1) and organic cation transporter 1 (hOCT1). They conducted an in vitro study using kidney cells stably expressing hOAT1/hOCT1, which found that GCV uptake was about sevenfold and twofold higher than controls, respectively [15]. Further evidence comes from a study by Nomura et al. [16] who reported that expression levels of OAT1 in rat kidneys reached near-mature levels by postnatal day 14. The amount of OAT1 proteins at PNA 14 was approximately 5 times higher than that at PNA 0, and minimal changes occurred thereafter [16]. These findings support the interpretation that the increased CL of GCV observed in the later treatment period reflects developmental changes in OAT1-mediated tubular secretion.

Nephrogenesis in humans starts at 9 weeks of gestation, with approximately 80% of nephrons forming between 20 and 36 weeks, and completion typically occurring by 34–36 weeks [17]. As the patient in this case was born at 27 weeks, renal development was likely incomplete at birth. In a previous study, Trang et al. [18] studied term infants with a mean PNA of 18. They reported mean GCV clearance of 0.213 L/hr/kg, which is similar to the GCV clearance of 0.219 L/hr/kg estimated at PNA 78 in the present study. This phenomenon is known as “catch-up” growth [19], which is characterized by rapid development of preterm infants, enabling them to attain the same developmental level as term infants at a certain point. Based on these observations, we hypothesize that catch-up growth also occurs in renal function. This should be considered when determining dosing regimens for renally excreted drugs in preterm neonates.

There are several limitations that must be acknowledged. First, the PPK parameters [9] used in this study were obtained from a study of 24 infants aged 8–34 days after birth and weighing 1.9–4.4 kg. The characteristics of this population differ from the present study. Second, the F value (conversion of VGCV to GCV) may not be constant during development, because the mRNA expression of esterase 1 and esterase 2 increases by approximately fivefold and fourfold, respectively, within 6 months after birth [20, 21]. Finally, it was difficult to perform adequate sampling because of the risk of hemolytic anemia due to frequent blood sampling.

## Conclusion

Our findings suggest that dose adjustment of GCV in preterm infants should be based not only on body weight but also on renal function.

## Data Availability

The datasets used and/or analyzed during the present study are available from the corresponding author on reasonable request.

## Abbreviations

CMV: Cytomegalovirus
GCV: Ganciclovir
VGCV: Valganciclovir
PPK: Population pharmacokinetic
Scr: Serum creatinine clearance
CL: Clearance
V: Volume of distribution
F: Bioavailability
Ka: Absorption rate constant
WT: Body weight
PNA: Postnatal age
hOAT1: Human organic anion transporter 1
hOCT1: Human organic cation transporter 1
Cr_u_: Urine creatinine concentration
V_u_: Total urine volume
S_cr_: Serum creatinine concentration
T: Sampling time
BSA: Body surface area
